# The Millet of the Matter: Archeobotanical Evidence for Farming Strategies of Western Han Dynasty Core Area Inhabitants

**DOI:** 10.3389/fpls.2022.929047

**Published:** 2022-06-30

**Authors:** Jingwen Liao, Ming Li, Edward Allen, Wuhong Luo, Pengfei Sheng

**Affiliations:** ^1^Department of Archaeological Sciences, Faculty of Archaeology, Leiden University, Leiden, Netherlands; ^2^Department for the History of Science and Scientific Archaeology, University of Science and Technology of China, Hefei, China; ^3^Hanyang Mausoleum Museum, Xi’an, China; ^4^Ministry of Education of the People’s Republic of China (MOE), Laboratory for National Development and Intelligent Governance, Department of Cultural Heritage and Museology, Fudan University, Shanghai, China; ^5^Institute of Archaeological Science, Fudan University, Shanghai, China; ^6^Center for the Belt and Road Archaeology and Ancient Civilizations, Fudan University, Shanghai, China

**Keywords:** paleoethnobotany, phytolith, foxtail millet, spread of wheat, buried model granaries, Han dynasty

## Abstract

Despite decades of investigation, consensus has yet to be reached on when and where wheat replaced millet as the primary crop in the core regions of early Imperial China. Previous studies have suggested that wheat cultivation likely became widespread prior to or during the Han Dynasty (202 BC–AD 220). Here, we tested this hypothesis by applying archeobotanical tools to plant remains found in five pottery model granaries (*cang*) entombed in a newly excavated late Western Han burial at the Longzaocun cemetery in the Guanzhong Basin. This analysis allowed us to explore the extent of wheat expansion and agricultural strategies in the heartland of early dynastic China. Macro- and micro-botanical evidence shows that the Longzaocun residents consumed two kinds of crops: foxtail and common millet. Combining these findings with previous studies, we argue that millet-based multi-crop farming dominated the regional agricultural system during the Western Han Dynasty (202 BC-AD 8) and analyze the political and cultural motivations for the Han people’s usage on millet crops from the burial concepts and fiscal systems. Echoing previous studies, we argue that millets remained the most valuable subsistence food for inhabitants of the Loess Basins in the Han core, and that wheat was not cultivated on a large scale in this area during the Western Han Dynasty.

## Introduction

The temporal and spatial distribution of ancient farming and its underlying dynamics are key issues for understanding past processes of human social development (Bellwood, 2005; Crawford, 2006). Relying on extensive archeobotanical, stable isotope, DNA, and other scientific archeological approaches, the central regions of global agricultural origins and main routes of crop dispersion during the Holocene have been outlined for the Chinese case (Zhao, 2019a). In China, considerable archeobotanical studies over the past two decades have made significant contributions in revealing the origin and development of Neolithic agriculture (Zhao, 2011a,b). Adaptations to global climate change during the terminal Pleistocene and initial Holocene precipitated the domestication of two Asian millet crops in the Yellow River valley at about 10,000 BP, while dryland farming dominated by millet cultivation was established in northern China at around 5,000 BP (Lu et al., 2009a; Yang et al., 2012; Zhao, 2020a,b). Despite these understandings of the prehistoric Chinese agricultural economy, research on agricultural dynamics in the subsequent dynastic period has been considerably lacking.

The Han Dynasty (202 BC-AD 220) was one of the most prosperous of the unified dynasties of Chinese history and at its time the most advanced civilization on the planet (Jian, 2019). An expanding iron smelting industry greatly improved the efficiency of agricultural production, and the small-scale peasant economy established in this period became the social and economic backbone of northern China for the next two millennia (Zhang, 1987; Huang, 2007). The Han government also attached great importance to its trade with Western peoples and polities, pioneering the Silk Road and bringing in grapes, walnuts, carrots, pomegranates, spices, and additional cash crops throughout this period (Min, 1991; Shi, 2014). Agricultural strategy and dynastic development clearly go hand-in-hand, at the same time offering a contribution to the archeological and historical research on the culture of major crops across the great length of Chinese history.

In historical documents, the primary Han dynasty farming package, known as the“*five grains*,” can be traced back to the *Analects of Confucius* (论语) (Shen, 1998). Yet there is no unified explanation for the composition of “*five grains*” in ancient documents, while some even believe a number higher than five was meant “*six grains*” or “*nine grains*” (Shen, 1998; Song, 2002). Harnessing the evidence in the archeological record, it is certain that foxtail millet (*Setaria italica*), common millet (*Panicum miliaceum*), rice (*Oryza sativa*), wheat (*Triticum aestivum*), soybean (*Glycine max*), and hemp (*Cannabis sativa*) were already consumed in the Han Dynasty (Liu, 2005, 2016; Zheng, 2021). Of these, foxtail millet, common millet, rice, and soybean were all domesticated in China and cultivated for millennia, and only wheat was indisputably an exotic import (Zhao, 2019a).

Reported early wheat remains in China are concentrated along the middle and lower reaches of the Yellow River and in China’s northwest region (Zhao, 2015). Carbonized wheat remains show that wheat was introduced to China from West Asia prior to 5,000 BP (Dodson et al., 2013; Liu et al., 2017; Long et al., 2018; Chen et al., 2020; Zhou et al., 2020). Mentions of wheat are frequent in the Chinese historical literature (Zhao and Chang, 1999; Han, 2013). However, the process driving the switch from a millet-based agricultural strategy to an economy more focused on wheat production remains unclear.

Although much work remains to be done to enhance our understanding of the Han empire’s agricultural economy, existing textual records in Chinese suggest that wheat cultivation in the core area of the Western Han Dynasty (202 BC–AD 8), the Guanzhong Basin, was relatively lagging compared to the Central Plains (Zhao and Chang, 1999). In the pre-Han period, recent stable C, N isotope analysis of bones from Central Plains sites during the Eastern Zhou dynasty (770–206 BC) have suggested that millets were the staple food of the nobility, while urban commoners consumed a considerable amount of wheat under considerable subsistence pressure (Zhou et al., 2017, 2019, 2021; Tian and Zhou, 2020; Zhou, 2020). Isotopic evidence has also indicated that wheat farming was promoted in the Shanxi area prior to the Han Dynasty (Tang et al., 2018), while other studies argue that rotation of summer millet and winter wheat may have been implemented in the Central Plains by the late Western Han (Zhao, 2020). These new findings have forced archeologists to reconsider regional and class differences in crop choice in the northern regions of early imperial China. In the present day, wheat has overwhelmingly replaced millet as the dominant crop species in agricultural production in northern China, but scholars remain uncertain exactly when and where it impacted the traditional millet-based agricultural pattern and exerted its subsequent profound impact on the heartland of early dynastic China.

**FIGURE 1:**
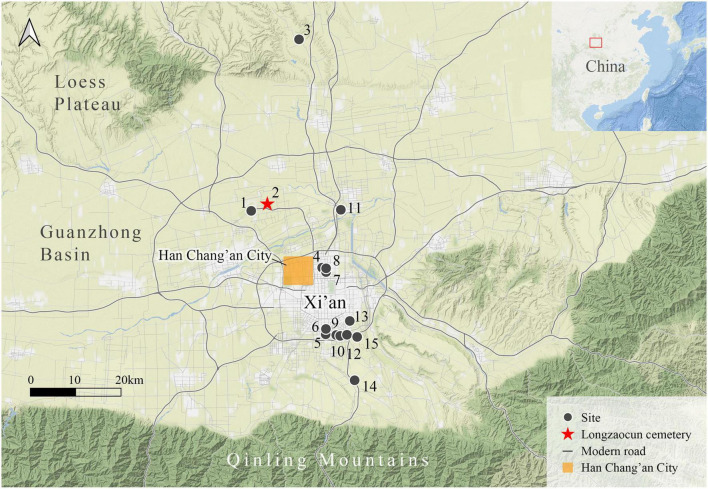
Location of the Longzaocun cemetery and other related archeological sites around Han Chang’an City in Xi’an. (1) Jichang; (2) Longzaocun; (3) Guandao; (4) Ronghai; (5) Shijia; (6) Shiyou; (7) Hairong; (8) Jiaoxiao; (9) Yannan; (10) Quchun; (11) Guangming; (12) Yanhu; (13) Ligong; (14) Xizha; (15) Sanzhao.

In the present study, we focus on newly generated data from an archeobotanical study on plant specimens recovered from five pottery model granaries buried containing an individual dated to the late Western Han Dynasty found at the Longzaocun cemetery (34°27′26.8″N, 108°48′21.7″E, Figure 1) in the Guanzhong Basin. This allows us to further examine the hypothesis of large-scale cultivation of wheat crops in the core political regions of early dynastic China. Our data is combined with existing archeological data from Han burials around modern-day Xi’an (Zhao, 2009; Zhang et al., 2013; see Figure 1, detailed in Supplementary Table 1). Our study provides a novel insight for understanding the essential agricultural strategies in the densely populated capital region at the dawn of Imperial China, circa 2,000 BP.

## Materials and Methods

**FIGURE 2:**
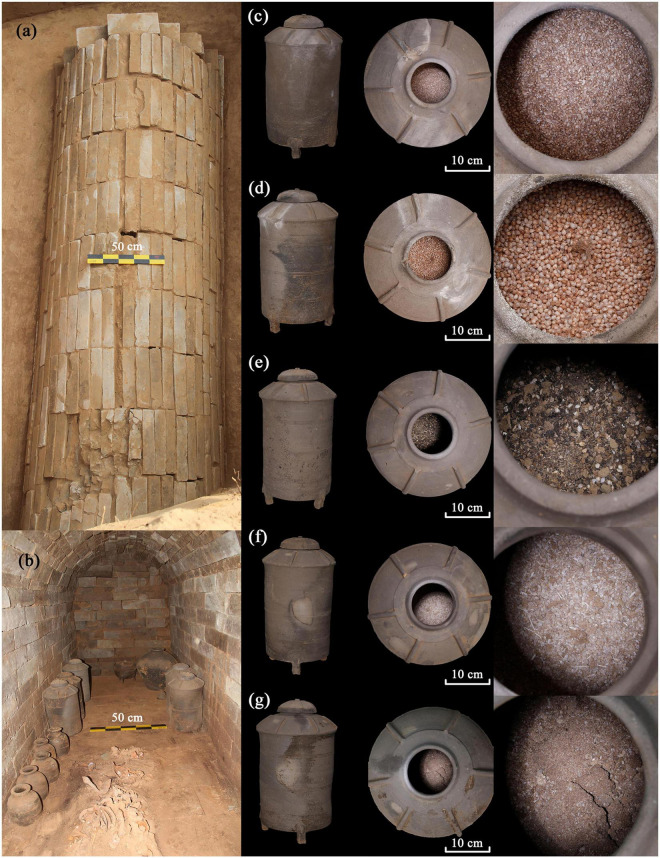
M111 tomb at the Longzaocun cemetery, **(a)** overhead photograph of the tomb; **(b)** interior photograph of M111 and buried objects, **(c–g)** photographs of pottery model granary and plant remains.

**FIGURE 3:**
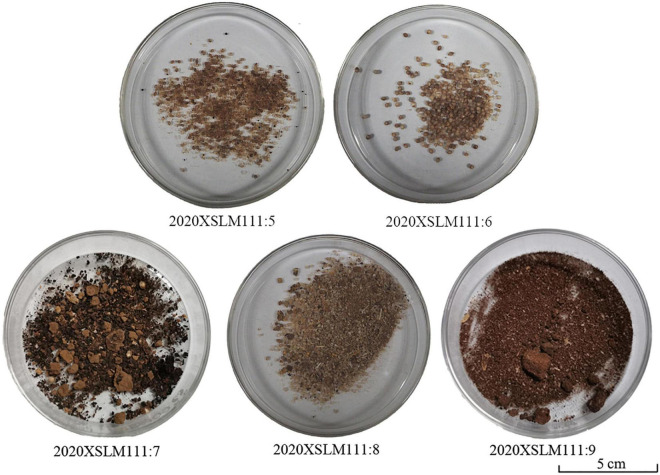
Study samples collected from the Longzaocun cemetery M111.

A total of five pottery model granary samples were collected from the brick tomb M111 (Figure 2) at Longzaocun cemetery. Based on the different degrees of preservation of the plant remains among the study samples (Figure 3), we selected macro-botanical methods and phytoliths and starch grains analyses to identify the relevant plant species. Specifically, three samples (M111: 5, M111: 6, M111: 8), dominated entirely by visible macro-botanical remains recovered from three model pottery granaries, were identified with a Nikon SMZ800N stereomicroscope at the Institute of Archeological Science, Fudan University. Our plant nomenclature followed the guidelines in TROPICOS^1^. All plant remains are currently stored in the Department of Cultural Heritage and Museology, Fudan University, Shanghai. Two additional soil samples without visible macro-botanical fossils, contained in the other two pottery granaries (M111: 7, M111: 9) were tested through phytolith and starch grains analysis, in order to determine the nature of these grain remains. These tests were used 3 mg samples and were performed at the Laboratory of Bio-Archeology at the University of Science and Technology of China in Hefei.

Starch grain analysis was first carried out with 5% (NaPO_3_)_6_ (Sangon Biotech, Shanghai) for anti-flocculation treatment. After reducing the dispersion of sediment samples, 10% HCI (Sinopharm Chemical Reagent Co., Ltd.) was mixed to remove carbonate impurities. Following this, a heavy liquid (CsCl, a density of 1.89, Sinopharm Chemical Reagent Co., Ltd.) was used to extract starch grain by L535-1 Floor-type Low Speed Macro Centrifuge, Cence, at 1,000 rpm in 8 min, 25°C. Phytolith analysis was performed on all samples following the procedures outlined by Piperno and Runge, with slight modifications (Piperno, 1988; Runge, 1999). First, before 10% HCI (Sinopharm Chemical Reagent Co., Ltd.) and 30% H_2_O_2_ (Sinopharm Chemical Reagent Co., Ltd.) were treated to remove organic matter and carbonates, 5% (NaPO_3_)_6_ (Sangon Biotech, Shanghai) was also chosen to clean the clay. Phytoliths were then extracted using a heavy liquid (ZnBr_2_, a specific density of 2.35, Sinopharm Chemical Reagent Co., Ltd.).

Extracted starch grains and phytoliths were mounted on slides with 25% Glycerol (Sinopharm Chemical Reagent Co., Ltd.) and Canada balsam medium (Sinopharm Chemical Reagent Co., Ltd.) respectively. Each slide was observed and photographed under a Leica DM4500P polarizing microscope (200× and 630×). The identification and nomenclature of starches and phytoliths were based on the laboratory’s modern sample database and standard reference materials (Madella et al., 2005; Lu et al., 2009b; Neumann et al., 2019; Henry, 2020).

## Results

### Identification of Macro-Botanical Materials

A significant number of macro-botanical fossils of crops were recovered from pottery containers at Longzaocun cemetery M111. Two varieties of Asian millet taxa, including *Setaria italica* and *Panicum miliaceum* had been collected and identified. As can be seen in Figures 2c,d,f, these sampled macro-plant remains are mainly husks from foxtail millet or common millet.

### Identification of Phytoliths and Starch Grains

**FIGURE 4:**
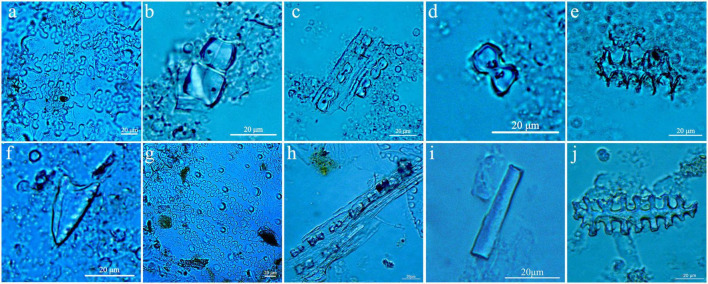
Primary phytolith types found at Longzaocun cemetery M111, **(a)** Ω-type from foxtail millet; **(b)** long-saddle; **(c)** vertically arranged bilobates with rounded ends; **(d)** bilobate; **(e)** elongate-echinate; **(f)** acicular; **(g)** Ω-type; **(h)** vertically arranged bilobates with rounded ends; **(i)** smooth-elongate; **(j)** elongate-echinate.

Six types of phytolith were found in the M111: 7 samples. These included identifiable Ω-type phytoliths from the husks of foxtail millet (Figure 4a), long-saddle short cell phytolith from the Bambusoideae (Figure 4b), and vertically arranged bilobate short cells with rounded ends from stems and leaves from Panicoideae (Figure 4c). Other common phytoliths from unknown species were bilobate short cell (Figure 4d), elongate-echinate long cell (Figure 4e), and acicular (Figure 4f) in form.

Four types of phytolith were observed in the M111: 9 samples. These included identifiable Ω-type phytolith from the husks of foxtail millet (Figure 4g), vertically arranged bilobate short cells with rounded ends from stems and leaves from Panicoideae (Figure 4h), smooth-elongate long cell (Figure 4i), and elongate-echinate long cell (Figure 4j). No starch grain was observed in the two samples.

## Discussion

Previous studies have shown that millet-dominated agricultural production had formed across north China by the middle and late Neolithic periods (Zhao and He, 2006; Zhang et al., 2010; Liu et al., 2013; Liu, 2014; Wang et al., 2015; Zhong et al., 2015; Zhao, 2017, 2019b). With the establishment of the unified Han empire, centuries of war in northern China during the Spring and Autumn and Warring States periods (770–206 BC) came to a close, and social development gradually improved. As well documented, a single risk-tolerant millet agricultural production system could no longer provide for the needs of rapidly growing urban populations around Han Chang’an City (Bray and Zheng, 1982; Shang, 2003; Lu, 2014). Western Han emperors issued several edicts to promote the cultivation of wheat crops to solve problems of food shortage caused by this increased population pressure (Zhao and Chang, 1999). Yet, archeology still has little to say about the new agricultural strategies for such challenging conditions. By combining the results of scientific archeological research with the historical records, we re-examine the farming system chosen by the core area residents of the Western Han Empire (in modern-day Xi’an) in order to maintain and improve agricultural productivity.

Zhao has identified the macro-plant remains recovered from 45 pottery granary models buried with Han tomb owners in Xi’an (Zhao, 2009). His work shows that foxtail millet appeared with the greatest frequency (*n* = 20, 44%), followed by common millet (*n* = 13, 29%), soybean (*n* = 5, 11%), adzuki bean (*n* = 4, 8%), barley (*n* = 3, 7%), rice (*n* = 2, 4%), hemp (*n* = 2, 4%), wheat (*n* = 1, 2%), and Job’s tears (*n* = 1, 2%) (Zhao, 2009). Additional stable isotope analysis of 42 human samples from three Han dynasty cemeteries in the Guanzhong area (summarized in Supplementary Table 1) also revealed that the diets of individuals living around the Han capital city of Chang’an were significantly influenced by millet-based foods (Zhang et al., 2013). In this study, the frequency of foxtail millet was significantly higher than that of common millet, reaching 80%. Given the new and existing archeological evidence, we believe that a multi-crop cultivation system reached extensively across the heartland of the Western Han Dynasty. Specifically, we observe that foxtail millet still occupied a dominant position in regional agricultural production. Additionally, common millet should be considered a main supplementary cereal crop around Chang’an during the Han Dynasty.

It is worth noting that no wheat remains were recovered from the late Western Han burial of M111 at the Longzaocun cemetery, and that the unearthed proportion of wheat in the pottery models of granaries of other Han tombs in Xi’an was insignificant (see Supplementary Table 1). Previous research has demonstrated that a belief in the afterlife in Han times led to the construction of grave goods imitating real-life models (Luo, 2005; Liu, 2007). The pottery model granary was modeled on a real-life prototype (see Figure 2). Model granaries and other necessary equipment were placed in burials for the enjoyment of tomb owners in the afterlife (Zhou, 2003; Li, 2012). This unique funeral concept granted the model pottery granary an additional significance as not only a food container but also a reflection of the deceased’s view of wealth and a yearning for a prosperous afterlife in Han people’s ritual practices. It has been pointed out that food choice and value demonstrate people’s economic position and aspirations (Engels, 1942). The construction of the brick-chambered tomb required a certain amount of manpower and material resources, showing that the owner of the tomb M111 was most likely a wealthy civilian. The fact that foxtail millet and common millet crops were invariably buried inside the pottery model granary implies that wheat, a non-native crop to China, was not yet valued by the nobility during this period.

The most advantageous aspect of wheat is its much higher yield and staggered growing season by comparison to millet, which marked improved land utilization while avoiding flooding (Peng, 2010). However, existing evidence reveals that the status of this foreign crop, despite its obvious merits was still lower than that of millets in the Guanzhong basin even 2,000 years following its introduction to the central area of China. On the one hand, it has been argued arid natural climatic conditions in the Guanzhong region could not meet the water demand for wheat growth (Zhu, 1964; Jing and Hui, 2007). Although the large irrigation projects were constructed during the Han, it was likely that these projects merely relieved rather than fundamentally resolving the pressure of natural conditions on intensive agricultural production (Peng, 2010). On the other hand, the traditional consumption of grain-eating greatly affected the taste of wheat, making the latter less easily digested (Zeng, 2007). As Sadao (1992) suggested, only with the vigorous development of grinding tools during the Tang Dynasty (AD 618–907) was flour processing technology popularized in Guanzhong Basin.

In addition to the above-mentioned limitations of natural conditions and farming techniques, our new evidence allows us to argue that socio-political and cultural preferences also explain why wheat could not be popularized in the political center of the Han Dynasty. One way economic wealth feeds political power is through control over food, specifically through authority over the production of and access to food (Hastorf, 2017). Prior to the Tang Dynasty (AD 618–907), the main object of food taxation was millet, with levies on rice or wheat existing only in those areas where millet was not cultivated (Sadao, 1992). Millets retained a position of overriding importance within this political and social system. Salary rankings for Han officials, for example, were measured in millet (Zhang, 1996), further suggestion that greater amounts of millet and the resources to acquire this crop were in elite hands. To unify agricultural regulation and management, the Han government established a system of granary networks at different levels, in accordance with the distribution of administrative divisions and military garrisons across the empire (Shao, 1998, 2005; Lv, 2012; Kim, 2014). Through the circulation and redistribution of food in the granaries, millet as a staple food was firmly controlled by the rulers and noble classes (Kim, 2014; Dong, 2015). In light of this system, the millet could be said to be, to some extent, the material basis for the strengthening of centralization and the target for political group control, hence enjoying a high status in the political economy of the country. We speculate that occupants of the Han heartlands as seen at Longzaocun placed greater store on millets as a result of this strengthening of a millet-dominated view of wealth and power under the Han taxation, granaries and wage rankings, and that these then manifested in the burial customs of the nobles during the late Western Han Dynasty.

## Conclusion

This paper has provided new results from archeobotanical analysis of plant remains found in five pottery model granaries unearthed from a burial of the Western Han Dynasty at the Longzaocun cemetery burial M111, situated in the Guanzhong Basin. Foxtail millet and common millet were recovered and identified from these model granaries. Combining our finds with existing archeobotanical data in the surrounding region, we propose that millet-based agricultural production continued to dominate the heartland of the Chinese empire during the late Western Han Dynasty, although wheat and other crops may have emerged as a supplement within the overall agricultural economy. The lack of large-scale cultivation of wheat by late Western Han Dynasty core area dwellers is attributable to a popular view of life and death and the logistics of the Han government fiscal system. This report deepens the current understanding of agricultural production and crop usage ways in the political core area of dynastic China and also provides a political and cultural perspective on how millet represents wealth and power in the Guanzhong Basin at circa 2,000 BP. Further systematic multidisciplinary investigations of archeological materials from the Han through Tang dynasties are required in order to assess when, where, and how wheat crops changed the deep-rooted millet-based agricultural system in northern China.

## Data Availability Statement

The original contributions presented in this study are included in the article/Supplementary Material, further inquiries can be directed to the corresponding author.

## Author Contributions

PS conceived the idea for the study. ML performed and supervised the archeological work. JL and WL performed or supervised the wet laboratory work. JL, EA, and PS wrote and edited the manuscript. All authors contributed to the article and approved the submitted version.

## Conflict of Interest

The authors declare that the research was conducted in the absence of any commercial or financial relationships that could be construed as a potential conflict of interest.

## Publisher’s Note

All claims expressed in this article are solely those of the authors and do not necessarily represent those of their affiliated organizations, or those of the publisher, the editors and the reviewers. Any product that may be evaluated in this article, or claim that may be made by its manufacturer, is not guaranteed or endorsed by the publisher.

## Footnotes

1
http://www.tropicos.org/Home.aspx

## Supplementary Material

Supplementary Table 1 Macro-botanicaland isotopic data.
